# Sequence Set Design for a New LFM-PC Hybrid Modulated Radar Signal

**DOI:** 10.3390/s21155227

**Published:** 2021-08-02

**Authors:** Shiyi Li, Yuying Wang, Jindong Zhang, Daiyin Zhu

**Affiliations:** 1 Key Laboratory of Radar Imaging and Microwave Photonics, Ministry of Education, Nanjing University of Aeronautics and Astronautics, Nanjing 210016, China; wangyuying974@163.com (Y.W.); zhangjd@nuaa.edu.cn (J.Z.); zhudy@nuaa.edu.cn (D.Z.); 2 Leihua Electronic Technology Research Institute, Aviation Industry Corporation of China, Wuxi 214063, China

**Keywords:** hybrid modulated signal, ambiguity function, Doppler tolerance, signal optimization design, alternating direction method of multipliers

## Abstract

In this paper we study the code design problem of a new form of linear frequency modulation phase-coded (LFM-PC) hybrid signal with wide Doppler tolerance based on a range-Doppler discrete ambiguity function (DAF) to get better detection performance and anti-jamming capability. The DAF of the LFM-PC inter pulse signal is derived within the Doppler tolerance. Two optimization models are established. One is single pulse sequence design (SSD) for Doppler tolerance extension based on minimum integral normalized sidelobe level (INSL); the other is multi pulse sequence set design (MSSD) for signal orthogonality based on the minimizing sum of the normalized DAF sidelobe (NDAFSL) and discrete cross ambiguity function (DCAF). Two low-complexity signal optimization methods based on alternating direction method of multiplier (ADMM) are proposed, respectively. The simulation results show that the optimized signals have either wide Doppler tolerance or good orthogonal performance, and the optimization methods (i.e., SSD-ADMM and MSSD-ADMM) have the characteristics of fast convergence speed and low operation amount.

## 1. Introduction

In the last decade, high-speed processors have enabled radar systems to take full advantages of received signals to obtain sensible performance improvements in their detection and anti-jamming capability [1]. Meanwhile, modern multifunction phased array radar systems equipped with digital arbitrary waveform generators give the ability to generate high-accuracy, sophisticated, time-varying radar waveforms to achieve good anti-jamming performance and sensing objectives, such as improved detection performance with ideal ambiguity function (AF) shape [2,3]. As a consequence, in radar engineering, it has become crucial to optimize the transmitted waveform on a pulse-by-pulse basis to meet performance requirements, in terms of high detection possibility with good Doppler tolerance and low intercept probability with good orthogonal performance.

Linear frequency modulated (LFM) signal is well-known for its excellent characteristics, such as constant-envelope, easy generation, and good Doppler tolerance [4,5]. However, those anti-jamming methods based on LFM signal have poor performance, due to its simple and fixed form. Phase-coded (PC) signal (e.g., constant modulus sequence set) is another widely used waveform in several areas, for its high resolution and ranging accuracy. However, on account of its Doppler sensitivity, PC signal is usually restricted to detecting targets whose velocity ranges are a priori known. Taking into account the advantages of LFM and PC signals, we obtain the LFM-PC hybrid modulation signal. As a result, Doppler tolerance is extended, compared with a PC signal, and the complexity of the signal form is greatly increased to achieve better anti-jamming ability in radar systems.

It must be pointed out that the traditional LFM-PC signal is linear-frequency modulated within the pulse and coded by the sequence set between the pulses. In order to obtain better anti-jamming ability and wider Doppler tolerance, we investigate a new form of LFM-PC signal that is modulated by linear frequency and sequence set simultaneously within the pulse. Furthermore, considering the quantification of the digital to analog converter (DAC) of the radar waveform generator, we should optimize the constant modulus sequence set with a discrete phase constraint. In this paper, we pay attention to the unimodular sequence design of coherent waveform-agile radar on a predefined area of range-Doppler discrete ambiguity function (DAF) within the Doppler tolerance. In order to obtain better detection and orthogonal performance, the research was carried out in two scenarios; the first was a single pulse sequence design based on minimum INSL; the second was a multi pulse sequence set design based on minimizing the sum of NDAFSL and DCAF.

Over the last few decades, extensive research has investigated transmit waveform design in the radar research area. The optimal radar waveform is synthesized based on different performance objectives. Some studies have focused on shaping the AF of agile waveforms in sensing. In the works [6,7,8,9,10,11,12,13], the dynamic selection of waveforms for target tracking was considered, and the optimal waveform parameters were derived for tracking target motion using a linear/nonlinear observations model with detection in a clutter/clutter-free environment. In [14], a kind of efficient gradient method was proposed for designing AFs for multistatic primary surveillance radar systems. Kerahroodi [15,16] applied the coordinates down (CD) to the optimization design of a PC signal. Agile waveform can also be designed from the viewpoint of orthogonality. Orthogonal waveforms are widely used in multiple input multiple output (MIMO) radar systems [17] and anti-spoof jamming technology [18]. In the literature [19], the orthogonal waveform of MIMO radar is designed by constrained nonlinear programming based on the optimization criteria of minimizing the peak correlation sidelobe. Khan [20] and Huang [21] et al. considered the influence of Doppler frequency when designing orthogonal waveforms. Majumder [22] proposed to use Walsh orthogonal code to realize intra pulse phase-coded modulation. Wu Yue [23] used a LFM-PC signal to design an orthogonal waveform based on autocorrelation and cross-correlation AF. In [24,25], a CA-New (CAN) algorithm and a multi-CAN algorithm were proposed for peak sidelobe level (PSL) minimization and orthogonal waveform optimizing, respectively.

In the aforementioned works, the sequences designed by the methods mentioned above are arbitrary phase-coded signals. If we simply set the continuous phases to their closest discrete phase, the sequence performance will deteriorate drastically. To generate a sequence with a discrete phase set, many methods have been proposed and can be divided into two categories: the first is to use existing codes, such as Barker code or M code; the second is to use an intelligent search method to find the desired sequence. Genetic algorithm (GA) [26,27] is a search heuristic that is inspired by Charles Darwin’s theory of natural evolution. In [28], a general and complicated problem of discrete-phase sequence design has already been addressed, and a GA approach is exploited for minimizing ISL in code sequence design. Discrete-phase sequence design with GA has also been successfully applied for orthogonal frequency division multiplexing (OFDM) radar [29] and MIMO radars [30]. In this paper, the performance of a GA approach is compared with the proposed algorithms.

The alternating direction method of multipliers (ADMM) [31] is also a search algorithm that solves convex optimization problems by breaking them into smaller pieces, each of which are then easier to handle. It has recently found wide application in a number of areas. In [32], Boyd showed that the ADMM is well suited to large-scale convex optimization problems. Liang successfully used the ADMM method to design a unimodular sequence with low autocorrelation sidelobes [33] by solving a quadratic optimization problem with constraints. ADMM has also been successfully applied for radar in spectrally crowded environments [34] and MIMO radars [35,36]. Considering the high dimension in LFM-PC sequence set design problem and the decomposability and superior convergence properties of ADMM, we designed SSD-ADMM and MSSD-ADMM methods to solve this problem.

In this article, we propose an ADMM algorithm to design a discrete phase-coded sequence set on range-Doppler plane within Doppler tolerance. The rest of this work is organized as follows. Section 2 discusses the mathematical model and derives the DAF of LFM-PC signal. Section 3 proposes two optimization methods for optimizing the shape of DAF based on ADMM, i.e., SSD-ADMM and MSSD-ADMM. Section 4 demonstrates the proposed algorithms by extensive simulation experiments. Finally, concluding remarks and directions for future research are presented in Section 5.

## 2. LFM-PC Signal Model

In the following paragraphs the boldface upper case letters denote matrices; boldface lower case letters denote column vectors, and italics denote scalars. C denotes the complex field, and Z denotes the integer field. The superscripts ${\left(.\right)}^{T}$, ${\left(.\right)}^{*}$, and ${\left(.\right)}^{H}$ denote transpose, complex conjugate, and conjugate transpose, respectively. ⊗ denotes the Kronecker product of matrices. arg(.) denotes the argument of a complex number. argmax(.) and argmin(.) stand for the argument of the maximum and minimum, respectively. diag( . ) denotes the diagonal matrix formed by the entries, and $\left\|.\right\|$ indicates the Frobenius norm, <.,.> denotes inner product.

We assume a waveform agile radar transmits a coherent burst of *M* slow-time pulses. The baseband transmit signal can be formulated as the following general form:

(1) $$u\left(t\right)=\sum_{i=0}^{M-1}u_{i}\left(t-iT_{R}\right)$$

where $u_{i}\left(t\right)$ is the complex envelope of the *i*th transmit pulse, and $T_{R}$ is the pulse repetition interval (PRI). We assume that T is the pulse duration and that $T_{R}$ is much larger than T.

The complex envelope of the *i*th LFM-PC transmit pulse $u_{i}\left(t\right)$ is expressed as [4]:

(2) $$\begin{array}{cc} u_{i}\left(t\right)=e^{j\pi µt^{2}}\sum_{n=0}^{N-1}c_{i}\left(n\right)p_{n}\left(t\right), & 0\leq t\leq T \end{array}$$

where µ is the slope of frequency modulation; N is the sub-pulse length; $c_{i}\left(n\right)$ is the modulating code sequence of *i*th pulse, and $p_{n}(t)$ is an ideal rectangular shaping pulse of time length $T_{P}$, in which $T_{P}=\frac{T}{N}$ stands for the code element width.

Let, $c_{i}={\left[c_{i}\left(0\right),c_{i}\left(1\right),\cdots ,c_{i}\left(N-1\right)\right]}^{T}$ denote the modulating code sequence of the *i*th transmit subpulse, and code element $c_{i}\left(n\right)$ is modulated by discrete phase and expressed as

(3) $$c_{i}\left(n\right)=e^{j2\pi \frac{k_{i}\left(n\right)}{K}},\;n=0,1\cdots N-1,\;k_{i}^{}\left(n\right)\in \left[0,1,\cdot \cdot \cdot K-1\right]$$

where *K* denotes the number of discrete phases.

According to the literature [37], the AFs of the *i*th subpulse is expressed as:

(4) $$\chi_{i}\left(\tau ,f\right)=\int_{-\infty }^{\infty }u_{i}\left(t\right)u_{i}^{*}\left(t+\tau \right)e^{j2\pi ft}dt$$

where τ is the time delay, and f denotes the Doppler frequency shift. Substituting (2) into the definition of ambiguity function in (4), we can obtain:

(5) $$\chi_{i}\left(\tau ,f\right)=e^{-j\pi µ\tau^{2}}\sum_{n=0}^{N-1}\sum_{m=0}^{N-1}c_{i}\left(n\right)c_{i}^{*}\left(m\right)\int_{-\infty }^{\infty }p_{n}\left(t\right)p_{m}\left(t+\tau \right)e^{j2\pi \left(f-µ\tau \right)t}dt$$

In order to derivate the discrete ambiguity function (DAF) of the *i*th transmit pulse, we suppose that there are $N_{S}$ sampling points within each code element and that the sampling period is $T_{S}=\frac{T_{P}}{N_{S}}$; the total number of sampling points in a pulse is $N^{\prime }=N\cdot N_{S}^{}$. The signal in (2) can be rewritten as:

(6) $$\begin{array}{cc} u_{i}\left(t\right)=e^{j\pi µt^{2}}\sum_{n=0}^{N^{\prime }-1}{c^{\prime }}_{i}\left(n\right){p^{\prime }}_{n}\left(t\right), & 0\leq t\leq T \end{array}$$

where $c_{i}{}^{\prime }={\left[c_{i}{}^{\prime }\left(0\right),c_{i}{}^{\prime }\left(1\right),\cdots ,c_{i}{}^{\prime }\left(N^{\prime }-1\right)\right]}^{T}=c_{i}⊗1_{Ns}$, $1_{Ns}={\left[1,1,1...1\right]}^{T}$ represents the *i*th code sequence after sampling; ${p^{\prime }}_{n}\left(t\right)$ indicates ideal rectangular pulse with the width $T_{S}$. Thus, AF in (5) can be rewritten as:

(7) $$\chi_{i}\left(\tau ,f\right)=e^{-j\pi µ\tau^{2}}\sum_{n=0}^{N^{\prime }-1}\sum_{m=0}^{N^{\prime }-1}{c^{\prime }}_{i}\left(n\right){c^{\prime }}_{i}^{*}\left(m\right)\int_{-\infty }^{\infty }{p^{\prime }}_{n}\left(t\right){p^{\prime }}_{m}\left(t+\tau \right)e^{j2\pi \left(f-µ\tau \right)t}dt$$

We set $\begin{array}{cc} \tau =kT_{S}, & -(N^{\prime }-1)\leq k\leq N^{\prime }-1 \end{array}$ to discretize the delay time axis, and we also set $\begin{array}{cc} f=µpT_{S}, & -(N^{\prime }-1)\leq p\leq N^{\prime }-1 \end{array}$ to discretize the Doppler shift axis and obtain:

(8) $$\chi_{i}\left(kT_{S},µpT_{S}\right)=e^{j\pi µT_{S}^{2}\left(p-k-k^{2}\right)}\mathrm{sinc}\left[µT_{S}^{2}(p-k)\right]\sum_{n=0}^{N^{\prime }-1}{c^{\prime }}_{i}\left(n\right){c^{\prime }}_{i}^{*}\left(n+k\right)e^{j2n\pi µT_{S}^{2}\left(p-k\right)}$$

We write $\chi_{i}\left(kT_{S},µpT_{S}\right)$ as $\chi_{i}\left(k,p\right)$ for short, and define:

(9) $$\varsigma_{k,p}=e^{j\pi µT_{S}^{2}\left(p-k-k^{2}\right)}\mathrm{sinc}\left[µT_{S}^{2}(p-k)\right]$$

(10) $$D_{k,p}=diag\left(1,e^{j2\pi µT_{S}^{2}\left(p-k\right)},\cdots ,e^{j2\left(N^{\prime }-1\right)\pi µT_{S}^{2}\left(p-k\right)}\right)$$

(11) $$J_{k}={\left(\begin{array}{cc} 0_{k\times (N^{\prime }-k)} & 0_{k\times k}\\ I_{N^{\prime }-k} & 0_{(N^{\prime }-k)\times k} \end{array}\right)}_{N^{\prime }\times N^{\prime }}$$

(12) $$H\left(k,p\right)=\varsigma_{k,p}J_{k}D_{k,p}$$

Then the DAF of LFM-PC signal can be abbreviated as:

(13) $$\chi_{i}\left(k,p\right)={c^{\prime }}_{i}^{H}H\left(k,p\right){c^{\prime }}_{i}$$

The above range-Doppler DAF defined on discrete range and Doppler plane is widely used in the designing of radar waveforms. Since the shape of DAF is related to the modulation sequence codes, $c_{i}{}^{\prime }$, then we can shape the DAF by optimizing $c_{i}{}^{\prime }$ within the Doppler tolerance on discrete range and Doppler plane to satisfy the requirement in sensing objectives.

## 3. Optimization Process

The ADMM algorithm is a computational framework for solving optimization problems, which is suitable for solving convex optimization problems with distributed structure. ADMM decomposes a large global problem into several smaller, easier to solve local subproblems by decomposing the coordination process and obtains the solution of the large global problem through the solution of the coordination subproblem.

The expression of ADMM for solving problems is expressed as [31]:

(14) $$\begin{array}{l} \begin{array}{cc} \min & f\left(x\right) \end{array}+g\left(z\right)\\ \begin{array}{cc} s.t. & Ax+Bz=c \end{array}, \end{array}$$

It is transformed to an augmented Lagrangian function:

(15) $$L_{\rho }\left(x,z,\lambda \right)=f\left(x\right)+g\left(z\right)+\lambda^{T}\left(Ax+Bz-c\right)+\frac{\rho }{2}{\left\|Ax+Bz-c\right\|}_{2}^{2},$$

where ρ is the penalty coefficient.

Set u=λ/ρ, then the solution steps can be expressed as follows [38]:

(16) $$x^{\left(t+1\right)}=\underset{x}{\arg\min}\left(f\left(x\right)+\frac{\rho }{2}{\left\|Ax+Bz^{\left(t\right)}-c+u^{\left(t\right)}\right\|}_{}^{2}\right),$$

(17) $$z^{\left(t+1\right)}=\underset{z}{\arg\min}\left(g\left(z\right)+\frac{\rho }{2}{\left\|Ax^{\left(t+1\right)}+Bz-c+u^{\left(t\right)}\right\|}_{}^{2}\right),$$

(18) $$u^{\left(t+1\right)}=u^{\left(t\right)}+Ax^{\left(t+1\right)}+Bz^{\left(t+1\right)}-c.$$

### 3.1. Single Pulse Sequence Design

#### 3.1.1. Optimization Model

In single pulse sequence design, the DAF of LFM-PC signal in (13) can be simplified as:

(19) $$\chi \left(k,p\right)={c^{\prime }}_{}^{H}H\left(k,p\right){c^{\prime }}_{}$$

The amplitude of the main peak of DAF can be expressed as:

(20) $$\left|\chi \left(k_{0}^{},p\right)\right|=\left|{c^{\prime }}_{}^{H}H\left(k_{0}^{},p\right){c^{\prime }}_{}\right|=\underset{k}{arg\max}\left|{c^{\prime }}_{}^{H}H\left(k,p\right){c^{\prime }}_{}\right|,$$

where $-(N_{S}-1)\leq p\leq (N_{S}-1)$; when Doppler shift p is given, $\left|\chi_{}\left(k_{0}^{},p\right)\right|$ is a fixed value. According to the properties of AF, the maximum of $\left|\chi \left(k_{0}^{},p\right)\right|$ is at $\left|\chi \left(0,0\right)\right|$. When p≠0, $\left|\chi \left(k_{0}^{},p\right)\right|$ declines due to Doppler mismatch, the Doppler tolerance is defined as an area of the range-Doppler plane in which $\left|\chi \left(k_{0}^{},p\right)\right|$ decreases by no more than 3 dB, then the normalized discrete ambiguity function (NDAF) within the Doppler tolerance can be expressed as:

(21) $$\begin{array}{cc} \left|{\bar{\chi }}_{}\left(k,p\right)\right|={\frac{{c^{\prime }}_{}^{H}H\left(k,p\right){c^{\prime }}_{}}{\left|{c^{\prime }}_{}^{H}H\left(k_{0}^{},p\right){c^{\prime }}_{}\right|}}_{}, & -\left(N^{\prime }-1\right)\leq k\leq \left(N^{\prime }-1\right),-N_{DT}\leq p\leq N_{DT} \end{array},$$

where $N_{DT}^{}<(N_{S}^{}-1)$ indicates the boundary of Doppler frequency corresponding to Doppler tolerance.

Define the optimization area of NDAF on discrete range and Doppler plane as:

(22) $$\Psi =\left[\left(k,p\right)\left|N_{S}^{}\leq \left|k\right|\leq N^{\prime }\right.-1,0\leq \left|p\right|\leq N_{DT}^{}\right]$$

and the integral normalized sidelobe level (INSL) of this area can be expressed as:

(23) $$\mathrm{INSL}=\sum_{k,p\in \Psi }\frac{{\left|{c^{\prime }}_{}^{H}H\left(k,p\right){c^{\prime }}_{}\right|}_{}^{2}}{{\left|{c^{\prime }}_{}^{H}H\left(k_{0}^{},p\right){c^{\prime }}_{}\right|}_{}^{2}}$$

In order to improve the detection performance of radar in the case of Doppler detuning, the INSL of optimization area need to be minimized, and the optimization problem can be expressed as:

(24) $$\begin{array}{ll} P_{INSL}:\;\underset{c}{\min} & \sum_{k,p\in \Psi }\frac{{\left|{c^{\prime }}_{}^{H}H\left(k,p\right){c^{\prime }}_{}\right|}_{}^{2}}{{\left|{c^{\prime }}_{}^{H}H\left(k_{0}^{},p\right){c^{\prime }}_{}\right|}_{}^{2}}\\ \;\;s.t. & c_{n}=e^{j2\pi \frac{k_{n}}{K}},n=0,1,\cdots ,N-1,k_{n}\in \left[0,1,\ldots K-1\right]. \end{array}$$

#### 3.1.2. SSD-ADMM Optimization Method

We applying an ADMM framework to problem (24) by introducing the auxiliary variable z and constraint c=z, and obtain:

(25) $$\begin{array}{cc} P_{INSL-ADMM}:\underset{c}{\min} & \sum_{k,p\in \Psi }\frac{{\left|{c^{\prime }}_{}^{H}H\left(k,p\right){c^{\prime }}_{}\right|}_{}^{2}}{{\left|{c^{\prime }}_{}^{H}H\left(k_{0}^{},p\right){c^{\prime }}_{}\right|}_{}^{2}}+z^{H}z\\ s.t. & c=z\\ & z_{n}=e^{j2\pi \frac{k_{n}}{K}}\;,n=0,1,\cdots ,N-1,k_{n}\in \left[0,1,\ldots K-1\right]. \end{array}$$

Equation (24) is equivalent to (25), since $z^{H}z$ is a constant term under the above constraints. According to (15) and (25), and let $u=(\lambda_{r}+j\lambda_{i})/\rho$, so the augmented Lagrange equation is devised as:

(26) $$L_{\rho }\left(c,z,u\right)=\sum_{k,p\in \Psi }\frac{{\left|{c^{\prime }}_{}^{H}H\left(k,p\right){c^{\prime }}_{}\right|}_{}^{2}}{{\left|{c^{\prime }}_{}^{H}H\left(k_{0}^{},p\right){c^{\prime }}_{}\right|}_{}^{2}}+z^{H}z+\frac{\rho }{2}{\left\|c-z+u\right\|}^{2}-\frac{\rho }{2}{\left\|u\right\|}^{2}$$

where $\lambda_{r}$, $\lambda_{i}$, and ρ are Lagrange coefficients.

Let $c^{\left(t\right)}$ indicate the value of c after ADMM iterating t times (z and u correspond, represented as $z^{\left(t\right)},u^{\left(t\right)}$). Given the initial value $c_{}^{\left(0\right)},z_{}^{\left(0\right)},u_{}^{\left(0\right)}$, then the solution of the problem can be carried out according to the following steps:

(1) Update c. Suppose $z^{\left(t\right)},u^{\left(t\right)}$ as known quantities:
  (27) $$c^{\left(t+1\right)}=\underset{c}{\mathrm{argmin}}\left\{\sum_{k,p\in \Psi }\frac{{\left|{c^{\prime }}_{}^{H}H\left(k,p\right)c^{\prime }\right|}_{}^{2}}{{\left|{c^{\prime }}_{}^{H}H\left(k_{0}^{},p\right)c^{\prime }\right|}_{}^{2}}+\frac{\rho }{2}{\left\|c-z^{\left(t\right)}+u^{\left(t\right)}\right\|}^{2}\right\}.$$

To solve this problem, we need to transform (27) from the complex form to a real form Let, c˜′=Re(c′T)Im(c′T)T, z˜=Re(zT)Im(zT)T, c˜=Re(cT)Im(cT)T, u˜=Re(u˜T)Im(u˜T)T and A=Re(H)−Im(H)Im(H)Re(H), B=Im(H)Re(H)−Re(H)Im(H), then we can convert (27) to

(28) $${\tilde{c}}^{\left(t+1\right)}=\underset{\tilde{c}}{\mathrm{argmin}}\left\{\sum_{k,p\in \Psi }\frac{{\left(\tilde{c}{}^{\prime }{}^{T}A\left(k,p\right)\tilde{c}{}^{\prime }\right)}^{2}+{\left(\tilde{c}{}^{\prime }{}^{T}B\left(k,p\right)\tilde{c}{}^{\prime }\right)}^{2}}{{\left(\tilde{c}{}^{\prime }{}^{T}A\left(k_{0},p\right)\tilde{c}{}^{\prime }\right)}^{2}+{\left(\tilde{c}{}^{\prime }{}^{T}B\left(k_{0},p\right)\tilde{c}{}^{\prime }\right)}^{2}}+\frac{\rho }{2}{\left\|\tilde{c}-{\tilde{z}}^{\left(t\right)}+{\tilde{u}}^{\left(t\right)}\right\|}^{2}\right\}.$$

This problem is an unconstrained optimization problem, which can be solved with the quasi-Newton method [39]. In the following steps, we need to convert the real vector ${\tilde{c}}_{}^{\left(t+1\right)}$ back to the complex vector $c^{\left(t+1\right)}$.

(2) Update z. Suppose $c^{\left(t+1\right)},u^{\left(t\right)}$ as known quantities:
  (29) $$\begin{array}{cc} z^{\left(t+1\right)} & =\underset{z}{\mathrm{argmin}}\;z^{H}z+{\left\|c^{\left(t+1\right)}-z+u^{\left(t\right)}\right\|}^{2}\\ s.t. & z_{n}=e^{j2\pi \frac{k_{n}}{K}}\;,n=0,1,\cdots ,N-1,k_{n}\in \left[0,1,\cdot \cdot \cdot K-1\right]. \end{array}$$

By omitting the constant terms, the above problem is converted to

(30) zt+1=argmaxz Re[(c(t+1)+u(t))Hz]s.t.zn=ej2πknK,n=0,1,⋯,N−1,kn∈0,1,⋅⋅⋅K−1.

The elements z are independent of each other; therefore, they can be decomposed into N subproblems.

If taking $c_{n}^{\left(t+1\right)}+u_{n}^{\left(t\right)},z_{n}^{\left(t\right)}$ as vectors in the complex plane and ignoring the constant part, then we can obtain:

(31) $$\begin{array}{cc} z_{n}^{\left(t+1\right)} & \underset{k}{=\mathrm{argmax}}\cos\langle c_{n}^{\left(t+1\right)}+u_{n}^{\left(t\right)},z_{n}^{\left(t\right)}\rangle \\ s.t. & \begin{array}{cc} z_{n}=e^{j2\pi \frac{k_{n}^{}}{K}}, & k_{n}^{}\in \left[0,1,\cdot \cdot \cdot K-1\right]. \end{array} \end{array}$$

It is equivalent to find the integer $k_{n}(0\leq k_{n}<K)$, which make the angle between the vectors $z_{n}=e^{j2\pi k_{n}^{}/K}$ and $c_{n}^{\left(t+1\right)}+u_{n}^{\left(t\right)}$ minimum.

The solution $z_{n}^{\left(t+1\right)}$ is as follows:

(32) $$z_{n}^{\left(t+1\right)}=\exp\left(j2\pi \frac{\left\lfloor K\cdot \arg\left(c_{n}{}^{\left(t+1\right)}+u_{n}{}^{\left(t\right)}\right)/2\pi +\frac{1}{2}\right\rfloor }{K}\right).$$

(3) Update u.
  (33) $$u^{\left(t+1\right)}=u^{\left(t\right)}+c^{\left(t+1\right)}-z^{\left(t+1\right)}.$$

Therefore, the procedures of SSD -ADMM algorithm are as follows. Firstly, initialize variables $c^{\left(0\right)},z^{\left(0\right)},u^{\left(0\right)}$. Secondly, repeat steps (1) to (3), until the iteration stop condition is satisfied (the number of iterations reaches the upper limit or $\left|{\mathrm{INSL}}^{\left(t+1\right)}-{\mathrm{INSL}}^{\left(t\right)}\right|\leq \varepsilon$. ε is a smaller positive number as the convergence threshold). Thirdly, stop the iteration. The steps of solving the optimization model by SSD -ADMM are given in Table 1.

### 3.2. Multi Pulse Sequence Set Design

#### 3.2.1. Optimization Model

In multi pulse sequence set design, orthogonality is required for the transmit pulses to get better anti-jamming capability. Orthogonal performance can be measured by the cross ambiguity function (CAF), which is defined as:

(34) $$\chi_{ij}\left(\tau ,f\right)=\int_{-\infty }^{\infty }u_{i}\left(t\right)u_{j}^{*}\left(t+\tau \right)e^{j2\pi ft}dt$$

where $u_{i}\left(t\right)$ and $u_{j}\left(t\right)$ is the *i*th and *j*th transmit pulse, respectively. Accordingly, the discrete form of the CAF (DCAF) can be easily obtained as:

(35) $$\chi_{ij}\left(k,p\right)={c^{\prime }}_{j}^{H}H\left(k,p\right){c^{\prime }}_{i}$$

According to the orthogonal waveform design criteria, both the sidelobe of AF and CAF of the inter-pulse signals should be close to zero. Meanwhile, in order to improve the detection performance of the signal in the case of Doppler detuning, the optimization of DAF is also taken into account. Note that the definition of NDAF in (21) is not applicable for multi pulse design; we redefine the NDAF by an approximate formula of main peak of DAF.

In order to derive the amplitude of the main peak of DAF, we set $f=µ\tau \left(\tau >0\right)$, and assuming that $\left|\chi_{i}\left(\tau ,f\right)\right|$ is close to the main peak when the time delay τ is small enough, then (5) can be approximately transformed into a concise expression as:

(36) $$\begin{array}{c} \underset{\tau }{arg\max}\left|\chi_{i}\left(\tau ,f\right)\right|\approx \sum_{n=0}^{N-1}c_{i}\left(n\right)c_{i}^{*}\left(n\right)\int_{-\infty }^{\infty }p_{n}\left(t\right)p_{n}\left(t+\tau \right)dt\\ \;\;\;\;\;\;=\sum_{n=0}^{N-1}c_{i}\left(n\right)c_{i}^{*}\left(n\right)\int_{nT_{P}^{}}^{\left(n+1\right)T_{P}^{}-\tau }dt\\ \;\;\;\;\;\;=N\left(T_{P}^{}-\tau \right) \end{array}$$

Considering $f=µpT_{S}$, $T_{p}=T_{S}N_{s}$, the above equation of the main peak can also be transformed into discrete form as:

(37) $$\left|\chi_{i}\left(k_{0}^{},p\right)\right|\approx NT_{s}(N_{s}-\left|p\right|)-\left(N_{S}^{}-1\right)\leq p\leq \left(N_{S}^{}-1\right)$$

According to (5) and (37), the expression of NDAF can be derived as:

(38) $$\begin{array}{cc} {\bar{\chi }}_{i}\left(k,p\right)={c^{\prime }}_{i}^{H}\bar{H}\left(k,p\right){c^{\prime }}_{i}, & -\left(N^{\prime }-1\right)\leq k\leq \left(N^{\prime }-1\right),-N_{\mathrm{DT}}\leq p\leq N_{\mathrm{DT}} \end{array}$$

where $\bar{H}\left(k,p\right)=\frac{H\left(k,p\right)}{NT_{s}(N_{s}-\left|p\right|)}$, and $N_{\mathrm{DT}}^{}=\left\lfloor 0.293\cdot N_{S}^{}\right\rfloor$ is the boundary of Doppler tolerance on discrete range and Doppler plane, $\left\lfloor \cdot \right\rfloor$ stands for rounding.

Therefore, the optimization of the multi pulse sequence set of the LFM-PC signal can be carried out based on the minimization of the sum of NDAFSL and DCAF of the inter-pulse signals. The objective function can be expressed as:

(39) $$\Delta =\sum_{i}\sum_{k,p\in \Psi }{\left|{\bar{\chi }}_{i}\left(k,p\right)\right|}^{2}+\sum_{i\neq j}\sum_{k,p\in \Phi }{\left|\chi_{ij}\left(k,p\right)\right|}^{2}$$

where $\Psi =\left[\left(k,p\right)\left|N_{S}^{}\leq \left|k\right|\leq N^{\prime }\right.-1,0\leq \left|p\right|\leq N_{\mathrm{DT}}^{}\right]$ is the range of the NAF sidelobe within the Doppler tolerance, and $\Phi =\left[\left(k,p\right)\left|0\leq \left|k\right|\leq N^{\prime }\right.-1,0\leq \left|p\right|\leq N_{\mathrm{DT}}^{}\right]$ is the range of the Doppler tolerance on discrete range and Doppler plane.

#### 3.2.2. MSSD-ADMM Optimization Method

By applying ADMM to the quasi-Newtonian method to minimize the sum of NDAFSL and the CAF of the LFM-PC signal, and the optimization problem is:

(40) $$\begin{array}{c} P_{\Delta }:\underset{c}{\min}\sum_{i}\sum_{k,p\in \Psi }{\left|{c^{\prime }}_{i}^{H}\bar{H}\left(k,p\right){c^{\prime }}_{i}\right|}^{2}+\sum_{i\neq j}\sum_{k,p\in \Phi }{\left|{c^{\prime }}_{j}^{H}H\left(k,p\right){c^{\prime }}_{i}\right|}^{2}\\ \;s.t\;c_{i}\left(n\right)=e^{j2\pi \frac{k_{n}^{}}{K}}\;n=0,1,\cdots ,N-1;i=0,1,\cdots ,M-1;k_{n}^{}\in \left[0,1,\ldots K-1\right] \end{array}$$

Introducing auxiliary variable z and constraint c=z, we can obtain:

(41) $$\begin{array}{l} P_{\Delta -ADMM}:\underset{c}{\min}\sum_{i}\sum_{k,p\in \Psi }{\left|{c^{\prime }}_{i}^{H}\bar{H}\left(k,p\right){c^{\prime }}_{i}\right|}^{2}+\sum_{i\neq j}\sum_{k,p\in \Phi }{\left|{c^{\prime }}_{j}^{H}H\left(k,p\right){c^{\prime }}_{i}\right|}^{2}+z^{H}z\\ \;\;s.t.\;c=z\\ \;z_{i}\left(n\right)=e^{j2\pi \frac{k_{n}^{}}{K}},n=0,1,\cdots ,N-1;i=0,1,\cdots ,M-1;k_{n}^{}\in \left[0,1,\cdot \cdot \cdot K-1\right] \end{array}$$

where $c={\left[c_{0}^{T},c_{1}^{T}\ldots c_{M-1}^{T}\right]}^{T}$, $z={\left[z_{0}^{T},z_{1}^{T}\ldots z_{M-1}^{T}\right]}^{T}$. Note that problem (40) is equivalent to (41). According to ADMM and (41), and let $u=(\lambda_{r}+j\lambda_{i})/\rho$; the augmented Lagrange equation is written as:

(42) $$\begin{array}{c} L_{\rho }\left(c,z,u\right)=\sum_{i}\sum_{k,p\in \Psi }{\left|{c^{\prime }}_{i}^{H}\bar{H}\left(k,p\right){c^{\prime }}_{i}\right|}^{2}+\sum_{i\neq j}\sum_{k,p\in \Phi }{\left|{c^{\prime }}_{j}^{H}H\left(k,p\right){c^{\prime }}_{i}\right|}^{2}\\ +z^{H}z+\frac{\rho }{2}{\left\|c-z+u\right\|}^{2}-\frac{\rho }{2}{\left\|u\right\|}^{2} \end{array}$$

Let $c^{\left(t\right)}$ indicate the value of c After ADMM iterating t times (z and u are represented as $z^{\left(t\right)},u^{\left(t\right)}$). Given the initial value $c_{}^{\left(0\right)},z_{}^{\left(0\right)},u_{}^{\left(0\right)}$, then the solution of the problem can be carried out according to the following steps:

(1) Update c. Suppose $z^{\left(t\right)},u^{\left(t\right)}$ as known quantities:
  (43) $$\begin{array}{c} c^{\left(t+1\right)}=\underset{c}{\arg\;\min}\left\{\sum_{i}\sum_{k,p\in \Psi }{\left|{c^{\prime }}_{i}^{H}\bar{H}\left(k,p\right){c^{\prime }}_{i}\right|}^{2}\right.\\ \;\;\left.+\sum_{i\neq j}\sum_{k,p\in \Phi }{\left|{c^{\prime }}_{j}^{H}H\left(k,p\right){c^{\prime }}_{i}\right|}^{2}+\frac{\rho }{2}{\left\|c-z^{\left(t\right)}+u^{\left(t\right)}\right\|}^{2}\right\} \end{array}$$

This problem is an unconstrained optimization problem, which can be solved by the quasi-Newton method. First, we need to translate (43) from the complex form to a real form Let,c˜i=Re(c′iT)Im(c′iT)T, z˜=Re(zT)Im(zT)T, c˜=Re(cT)Im(cT)T, u˜=Re(u˜T)Im(u˜T)T, and A=Re(H)−Im(H)Im(H)Re(H), B=Im(H)Re(H)−Re(H)Im(H), then we can convert (43) to:

(44) $$\begin{array}{c} {\tilde{c}}^{\left(t+1\right)}=\underset{\tilde{c}}{\arg\;\min}\left\{\sum_{i}\sum_{k,p\in \Psi }{\left({\tilde{c}}_{i}^{T}\bar{A}\left(k,p\right){\tilde{c}}_{i}\right)}^{2}+{\left({\tilde{c}}_{i}^{T}\bar{B}\left(k,p\right){\tilde{c}}_{i}\right)}^{2}\right.\\ \;\;\left.+\sum_{i\neq j}\sum_{k,p\in \Phi }{\left({\tilde{c}}_{j}^{T}A\left(k,p\right){\tilde{c}}_{i}\right)}^{2}+{\left({\tilde{c}}_{j}^{T}B\left(k,p\right){\tilde{c}}_{i}\right)}^{2}+\frac{\rho }{2}{\left\|\tilde{c}-{\tilde{z}}^{\left(t\right)}+{\tilde{u}}^{\left(t\right)}\right\|}^{2}\right\} \end{array}$$

In the following steps, we need to convert the real vector ${\tilde{c}}_{}^{\left(t+1\right)}$ back to the complex vector $c_{}^{\left(t+1\right)}$.

(2) Update z. Suppose $c^{\left(t+1\right)},u^{\left(t\right)}$ as known quantities:
  (45) $$\begin{array}{cc} z^{\left(t+1\right)} & =\underset{z}{\arg\;\min}\;z^{H}z+{\left\|c^{\left(t+1\right)}-z+u^{\left(t\right)}\right\|}^{2}\\ s.t & z_{i}\left(n\right)=e^{j2\pi \frac{k_{n}^{}}{K}}n=0,1,\cdots ,N-1;i=0,1,\cdots ,M-1;k_{n}^{}\in \left[0,1,\cdot \cdot \cdot K-1\right] \end{array}$$

The above equation can be converted into

(46) zt+1=arg maxz Re(ct+1+ut)Hzs.tzin=ej2πknKn=0,1,⋯,N−1;i=0,1,⋯,M−1;kn∈0,1,⋅⋅⋅K−1

Note that problem (46) is a linear problem and that the elements of z are independent of each other; therefore, they can be decomposed into NM sub problems.

If taking $c_{i}^{\left(t+1\right)}\left(n\right)+u_{i}^{\left(t\right)}\left(n\right),z_{i}^{\left(t\right)}\left(n\right)$ as vectors in the complex plane and ignoring the constant part, then (46) can be transformed to:

(47) $$\begin{array}{cc} z_{i}^{\left(t+1\right)}\left(n\right) & \underset{k}{=\mathrm{argmax}}\cos\langle c_{i}^{\left(t+1\right)}\left(n\right)+u_{i}^{\left(t\right)}\left(n\right),z_{i}^{\left(t\right)}\left(n\right)\rangle \\ s.t & z_{i}\left(n\right)=e^{j2\pi \frac{k_{n}}{K}}\;k_{n}^{}\in \left[0,1,\cdot \cdot \cdot K-1\right] \end{array}$$

It is equivalent to find the integer k(0≤k<K), which make the angle between the vectors $z_{i}^{\left(t\right)}\left(n\right)=e^{j2\pi \frac{k}{K}}$ and $\left[c_{i}^{\left(t+1\right)}\left(n\right)+u_{i}^{\left(t\right)}\left(n\right)\right]$ minimal.

The solution of (47) is as follows:

(48) $$z_{i}^{\left(t+1\right)}\left(n\right)=\exp\left(j2\pi \frac{\left\lfloor K\cdot \arg\left[c_{i}^{\left(t+1\right)}\left(n\right)+u_{i}^{\left(t\right)}\left(n\right)\right]/2\pi +\frac{1}{2}\right\rfloor }{K}\right)$$

(3) Update u.
  (49) $$u^{\left(t+1\right)}=u^{\left(t\right)}+c^{\left(t+1\right)}-z^{\left(t+1\right)}.$$

Therefore, the procedures of MSSD-ADMM algorithm are as follows. Firstly, initialize variables $c^{\left(0\right)},z^{\left(0\right)},u^{\left(0\right)}$. Secondly, repeat steps (1) to (3) until the iteration stop condition is satisfied (the number of iterations reaches the upper limit or $\left|\Delta^{\left(t+1\right)}-\Delta^{\left(t\right)}\right|\leq \varepsilon$. ε is a smaller positive number as the convergence threshold). Thirdly, stop the iteration. The steps of solving the optimization model by MSSD-ADMM are given in Table 2.

### 3.3. Computational Complexity Analysis

In the ADMM algorithm, (43) dominates the main computation. We need to calculate the gradient of (43). Considering the subpulse length $N^{\prime }$, we can suppose Ψ contains $\alpha {N^{\prime }}^{2}$ points, where 0 < *α* < 1. Then, the computation of gradient contains

(50) $$\alpha \cdot {N^{\prime }}^{2}\cdot 2\cdot (N_{\mathrm{DT}}\cdot 8\cdot {N^{\prime }}^{3}+N_{\mathrm{DT}}\cdot 4\cdot {N^{\prime }}^{2})+2N_{\mathrm{DT}}N^{\prime }$$

times of real number multiplications and

(51) $$\alpha \cdot {N^{\prime }}^{2}\cdot 2\cdot (N_{\mathrm{DT}}\cdot 8\cdot {N^{\prime }}^{3}+N_{\mathrm{DT}}\cdot 4\cdot {N^{\prime }}^{2})+4N_{\mathrm{DT}}N^{\prime }$$

times of real number additions. Therefore, the computational complexity of ADMM algorithm is on the order of $o(N_{\mathrm{DT}}{N^{\prime }}^{5})$

We note that GA algorithm can also be successfully applied in discrete-phase sequence design problems. However, with increased variables in the LFM-PC sequent design problem, the efficiency of the GA algorithm is not satisfactory.

In each iteration of the GA algorithm, the number of variables in c is $N^{\prime }$, and for each variable $c_{n}$, we need to calculate the number $\alpha {N^{\prime }}^{2}$ locations in the DAF. The problem of $c_{n}$ in [31] has the computational complexity of $o(N_{\mathrm{DT}}{N^{\prime }}^{3})$.The total computational complexity of the GA algorithm is $o(N_{\mathrm{DT}}{N^{\prime }}^{6})$. Therefore, the computational complexity of tbe GA algorithm is higher than that of the ADMM algorithm.

## 4. Numerical Experiments

In this section, we provide two simulation examples to demonstrate the performance of the proposed method. In the following examples, it is assumed that the radar pulse width *T* is 10 µs and thatthe PRI equals 1 ms to ensure the unambiguity of the detect range. We also assume that the frequency modulation slope is $µ=6\times 10_{}^{12}$ to ensure the resolution for target detection. The coherent radar system transmits M pulses in a CPI and N subpulses in a pulse. In DAF image, the time delay axis is normalized by subpulse duration $T_{P}$, and the Doppler frequency shift axis is normalized by $1/T_{P}$. The convergence of the proposed algorithms will be tested by using randomly generated sequences in the initialization.

### 4.1. Single Pulse Sequence Design Case

In this example, in order to implement the algorithm, the Doppler tolerance needs to be calculated first. The PC signal is also taken into account for comparison. Let the code length of LFM-PC signals be N=60, the coding sequence consists of random codes. The code length and coding sequence of the PC signals are same as the LFM-PC signals. After 1000 instances of the Monte Carlo experiment, respectively, with modulation phase number *K* = 2/4/8, we calculate the Doppler tolerance of PC signals and LFM-PC signals. According to Table 3, the Doppler tolerance of the LFM-PC signal is obviously better than that of the PC signal regardless of the modulation phase number *K*.

To measure the effectiveness of optimization, we define Average Integral Normalized Sidelobe Level (AINSL) as:

(52) $$\mathrm{AINSL}=\frac{1}{2\left(N^{\prime }-N_{S}^{}\right)\left(2N_{DT}^{}+1\right)}\sum_{k,p\in \Psi }\frac{{\left|{c^{\prime }}_{}^{H}H\left(k,p\right)c^{\prime }\right|}_{}^{2}}{{\left|{c^{\prime }}_{}^{H}H\left(k_{0}^{},p\right)c^{\prime }\right|}_{}^{2}}.$$

where $\Psi =\left[\left(k,p\right)\left|N_{S}^{}\leq \left|k\right|\leq N^{\prime }\right.-1,0\leq \left|p\right|\leq N_{DT}^{}\right]$ represents the sidelobe range within the Doppler tolerance. The AINSLs of PC and LFM-PC signals are calculated respectively within Doppler tolerance by 1000 times of Monte Carlo experiments. It can be seen from Table 4 that the AINSLs of these two signals are almost the same.

**Figure 1:**
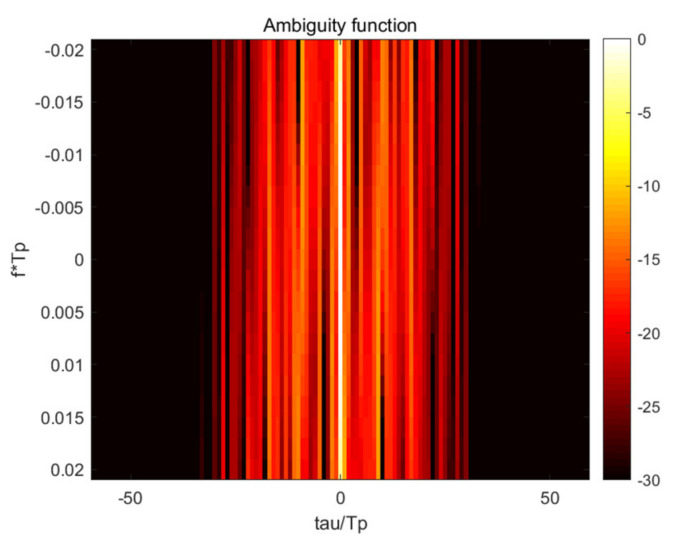
Ambiguity function before optimization.

**Figure 2:**
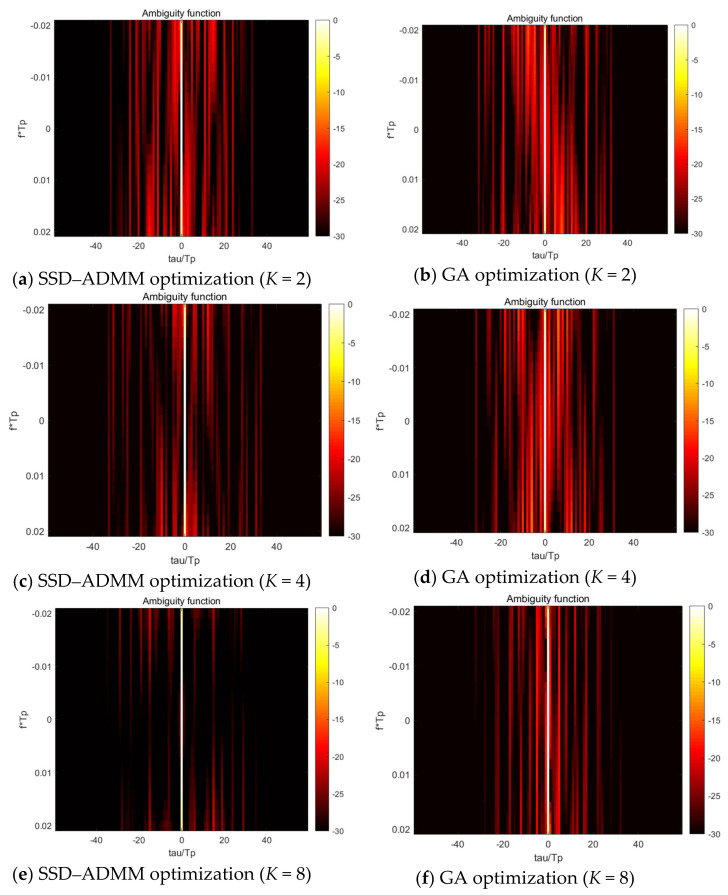
Ambiguity function after optimization.

In order to improve the detection performance of LFM-PC signals, the ambiguity function of LFM-PC signals was optimized by using the minimum INSL in Doppler tolerance. The upper limit of iteration number was set to 100 times, and the same initial signal was optimized by the above SSD-ADMM algorithm and GA algorithm, respectively. After iteration, the simulation results before and after optimization are shown in Figure 1 and Figure 2.

The AF can be seen as the matching filter output of a set of radar echo signals with same time delay and different Doppler frequency shift. As shown in the Figure 1, the weak target will be masked by the sidelobe of the strong target. So the sidelobe of AF must be suppressed and better detection performance can be obtained for closely spaced targets.

It can be seen from the Figure 1 and Figure 2 that the AFs after optimization are better than those before optimization, regardless of the SSD-ADMM algorithm or GA algorithm. But the optimization result of the SSD-ADMM algorithm is better than that of the GA algorithm.

**Figure 3:**
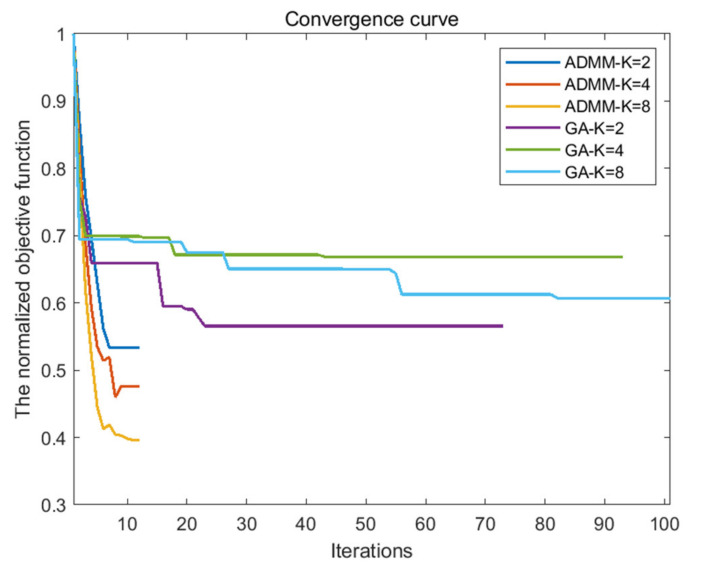
Comparison of convergence curve between SSD–ADMM and GA.

As can be seen from Table 4 and Table 5, the AINSL of LFM-PC signals optimized by the SSD-ADMM algorithm and the GA algorithm is significantly lower than that of the initial signal. Especially, with the increase in phase number, the optimization effect of the SSD-ADMM algorithm is more significant than that of the GA algorithm. At the same time, observing the iterative convergence curve of the optimization algorithm in Figure 3, we can find that SSD-ADMM algorithm converges faster and has better effect than the GA algorithm.

### 4.2. Multi Pulse Sequence Set Design Case

Considering the first and second term of the optimization model in (39), to measure the optimization performance of LFM-PC inter-pulse signal, auto correlation average side lobe, (AC-ASL) and cross correlation average level, (CC-AL) are defined as:

(53) $$\begin{array}{c} \mathrm{AC-ASL}=\frac{1}{2\left(N^{\prime }-N_{S}^{}\right)\left(2N_{\mathrm{DT}}^{}+1\right)\cdot M}\sum_{i}\sum_{k,p\in \Psi }{\left|{\bar{\chi }}_{i}\left(k,p\right)\right|}^{2}\\ \mathrm{CC-AL}=\frac{2}{\left(2N^{\prime }-1\right)\left(2N_{\mathrm{DT}}^{}+1\right)\cdot M(M-1)}\sum_{i\neq j}\sum_{k,p\in \Phi }{\left|\chi_{ij}\left(k,p\right)\right|}^{2} \end{array}$$

where $\Psi =\left[\left(k,p\right)\left|N_{S}^{}\leq \left|k\right|\leq N^{\prime }\right.-1,0\leq \left|p\right|\leq N_{\mathrm{DT}}^{}\right]$ is the range of the NAF sidelobe within the Doppler tolerance, and $\Phi =\left[\left(k,p\right)\left|0\leq \left|k\right|\leq N^{\prime }\right.-1,0\leq \left|p\right|\leq N_{\mathrm{DT}}^{}\right]$ is the range of the Doppler tolerance on discrete range and Doppler plane.

Set the code length of LFM-PC signals N=60, transmit pulse number M=3, and modulation phase number *K* = 2/4/8. Moreover, the original coding sequences consists of random codes. The aforementioned algorithms of MSSD-ADMM and GA are implemented on the same original signal, with an upper limit of iteration number of 50 times.

**Figure 4:**
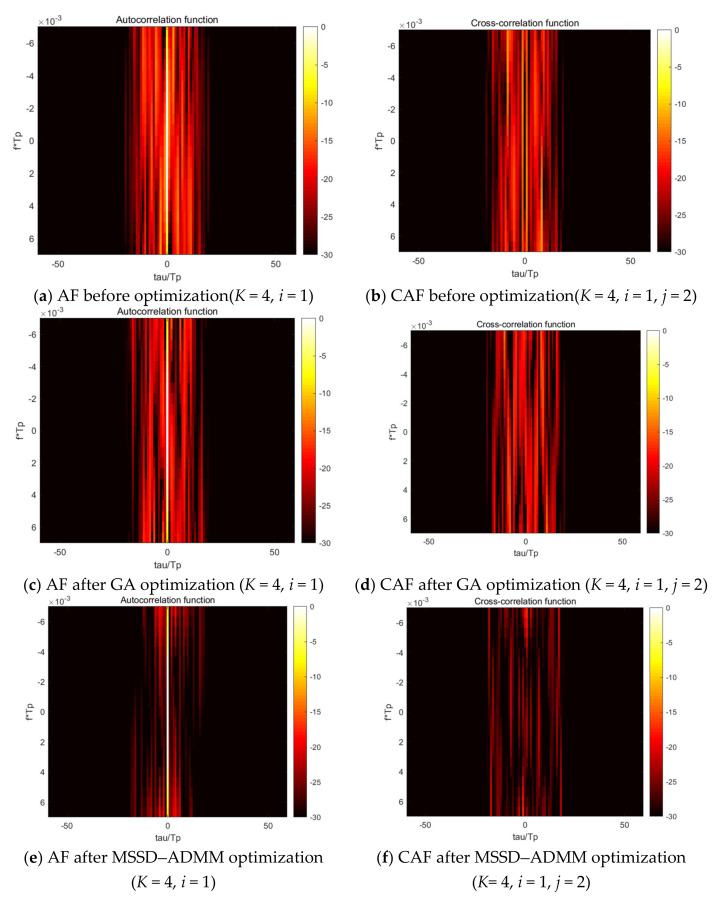
Diagram of AF and CAF before and after optimization.

Figure 4 shows a set of DAF and DCAF images of LFM-PC signals within the Doppler tolerance. It is observed that both the DAF side lobe (where τ≠0) and DCAF value of optimization signals are significantly lower than that of the original signal, regardless of the MSSD-ADMM algorithm or the GA algorithm. It also can be seen that the MSSD-ADMM method outperforms the GA algorithm.

Table 6 shows the AC-ASL and CC-AL metric of LFM-PC signals in different conditions. As can be seen from Table 6, compared with the original signal, the signals optimized by the MSSD-ADMM algorithm have significantly lower AC-ASL and CC-AL metric, and the optimization effect became better with the increase in the phase number *K*. As a comparison with the GA algorithm, the optimization effect is general, and with the increase of phase number K, the optimization effect decreases. Therefore, the LFM-PC signal optimized by the MSSD-ADMM algorithm has better waveform diversity gain and waveform isolation degree.

**Figure 5:**
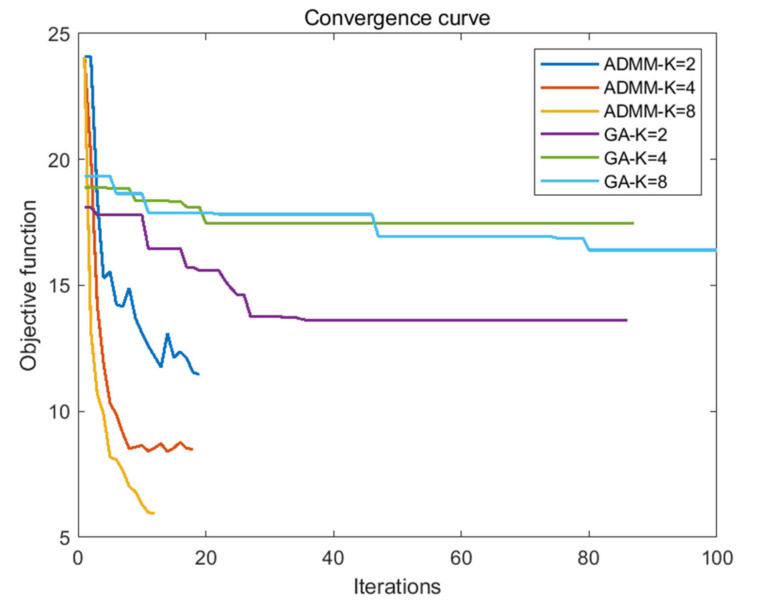
Comparison of convergence curve between MSSD–ADMM and GA.

By comparing the iteration convergence curve of the optimization algorithm in Figure 5 and the optimization algorithm time in Table 7, we can find that compared with the GA algorithm, the MSSD-ADMM algorithm has fewer iterations, faster convergence time, and better optimization effect.

As shown in the above two experiments, compared with the GA, ADMM has better optimization effect and faster convergence speed. The reasons for the significant difference between the two algorithms can be summarized as follows. Though GA is good at global search, it turns out in many applications to be inefficient in local search, especially at the later stages of evolution [40]. So the search speed of the algorithm is relatively slow, and more training time is needed to obtain a comparatively accurate solution. Moreover, in practice, with increased variables in a problem, GAs are prone to premature convergence [41]. That is to say, the optimization process stops at a stable point that does not represent a globally optimal solution. Whereas ADMM can be optimized locally through Lagrange [42], its convergence speed is much better than GAs, and its convergence effect is also better than GAs. Therefore, the simulation results are also better than GAs.

## 5. Conclusions

In this paper, the ADMM framework is used to optimize the LFM-PC signal and the orthogonal LFM-PC signal. At the same time, combined with the integer model of phase optimization, two new algorithms for LFM-PC optimization are proposed. From the perspective of the model, LFM-PC has better Doppler tolerance and better sidelobe performance than PC. Moreover, from the perspective of algorithm performance, compared with the GA algorithm, the proposed algorithm has faster convergence speed and less computation.

## Figures and Tables

**Table 1:** SSD-ADMM steps.

SSD-ADMM
(a) Set t as the times of iterations, initialize t=0 and given the initial value $c^{\left(0\right)},z^{\left(0\right)},u^{\left(0\right)}$; calculate $k_{0}^{}$ for each p;
(b) Use the quasi Newton method to solve the problem $c^{\left(t+1\right)}=\underset{c}{\mathrm{argmin}}\left\{\sum_{k,p\in \Psi }\frac{{\left|{c^{\prime }}_{}^{H}H\left(k,p\right)c^{\prime }\right|}_{}^{2}}{{\left|{c^{\prime }}_{}^{H}H\left(k_{0}^{},p\right)c^{\prime }\right|}_{}^{2}}+\frac{\rho }{2}{\left\|c-z^{\left(t\right)}+u^{\left(t\right)}\right\|}^{2}\right\}.$ (c) for n=0:N−1 $z_{n}^{\left(t+1\right)}=\exp\left(j2\pi \frac{\left\lfloor K\cdot \arg\left(c_{n}{}^{\left(t+1\right)}+u_{n}{}^{\left(t\right)}\right)/2\pi +\frac{1}{2}\right\rfloor }{K}\right).$ end; (d) $u^{\left(t+1\right)}=u^{\left(t\right)}+c^{\left(t+1\right)}-z^{\left(t+1\right)}$;
(e) t←t+1;
(f) If the convergence condition is satisfied, the algorithm is completed, otherwise it returns to (b)

**Table 2:** MSSD-ADMM steps.

MSSD-ADMM
(a) Set tas the times of iterations, initialize t=0,$\;\mathrm{and}\;\mathrm{give}\;\mathrm{the}\;\mathrm{initial}\;\mathrm{value}\;c^{\left(0\right)},z^{\left(0\right)},u^{\left(0\right)}$;
(b) Using quasi Newton method to solve the problem $c^{\left(t+1\right)}=\underset{c}{\mathrm{argmin}}\left\{\sum_{k,p\in \Psi }\frac{{\left|{c^{\prime }}_{}^{H}H\left(k,p\right)c^{\prime }\right|}_{}^{2}}{{\left|{c^{\prime }}_{}^{H}H\left(k_{0}^{},p\right)c^{\prime }\right|}_{}^{2}}+\frac{\rho }{2}{\left\|c-z^{\left(t\right)}+u^{\left(t\right)}\right\|}^{2}\right\}.$ (c) For i=0:M−1 for n=0:N−1 $z_{i}^{\left(t+1\right)}\left(n\right)=\exp\left(j2\pi \frac{\left\lfloor K\cdot \arg\left[c_{i}^{\left(t+1\right)}\left(n\right)+u_{i}^{\left(t\right)}\left(n\right)\right]/2\pi +\frac{1}{2}\right\rfloor }{K}\right)$ end; end (d) $u^{\left(t+1\right)}=u^{\left(t\right)}+c^{\left(t+1\right)}-z^{\left(t+1\right)}$;
(e) t←t+1;
(f) If the convergence condition is satisfied, the algorithm is completed, otherwise it returns to (b)

**Table 3:** Doppler tolerance.

	*K* = 2	*K* = 4	*K* = 8
PC	73.833 KHz	73.833 KHz	73.833 KHz
LFM-PC	202.6 KHz	201.8 KHz	204.9 KHz

**Table 4:** AINSL before optimization.

	*K* = 2	*K* = 4	*K* = 8
PC	−20.5436 dB	−20.0856 dB	−20.9821 dB
LFM-PC	−20.2248 dB	−20.2945 dB	−20.8219 dB

**Table 5:** AINSL after optimization.

	*K* = 2	*K* = 4	*K* = 8
SSD-ADMM	−26.8154 dB	−28.9818 dB	−31.4835 dB
GA	−25.9737 dB	−26.1257 dB	−28.2915 dB

**Table 6:** AC-ASL and CC-AL metric of LFM-PC (dB).

	K=2		K=4		K=8	
	AC-ASL	CC-AL	AC-ASL	CC-AL	AC-ASL	CC-AL
Original	−24.8040	−25.0624	−24.8334	−25.1614	−24.8560	−25.1963
GA	−28.0128	−26.8928	−26.6000	−26.0746	−26.8713	−26.3941
MSSD-ADMM	−28.7169	−27.6719	−29.0256	−29.9253	−31.1796	−30.8433

**Table 7:** Comparison of optimization time between MSSD-ADMM and GA.

	K=2	K=4	K=8
GA	41 min 04 s	38 min 25 s	45 min 02 s
MSSD-ADMM	10 min 23 s	12 min 45 s	13 min 30 s

## Data Availability

The datasets generated during the current study are not publicly available but are available from the corresponding author on reasonable request.
